# x‐Ray Structure of *Streptomyces avermitilis* Phospholipase D Reveals a Ca^2+^‐Stabilized Expanded Active‐Site Cleft Adapted for Phospholipid Binding

**DOI:** 10.1002/prot.70142

**Published:** 2026-04-30

**Authors:** Yoshiaki Yasutake, Tatsuya Hirata, Shunsuke Nomura, Kenji Konishi, Kazunari Yoneda, Shin‐ichi Sakasegawa, Haruhiko Sakuraba

**Affiliations:** ^1^ Biomanufacturing Process Research Center National Institute of Advanced Industrial Science and Technology (AIST) Sapporo Japan; ^2^ Nagase Diagnostics Co. Ltd. Shizuoka Japan; ^3^ Asahi Kasei Pharma Corporation Shizuoka Japan; ^4^ Department of Food and Life Sciences, School of Agriculture Tokai University Kumamoto Japan; ^5^ Biomanufacturing Process Research Center AIST Tsukuba Japan; ^6^ Department of Applied Biological Science, Faculty of Agriculture Kagawa University Kagawa Japan

**Keywords:** alkaline phosphatase, crystal structure, phospholipase D, *Streptomyces avermitilis*

## Abstract

Phospholipase D (PLD) catalyzes the hydrolysis of phospholipids to generate phosphatidic acid and free head groups such as choline. Among bacterial PLD enzymes, *Streptomyces chromofuscus* PLD (SchPLD), a member of the alkaline phosphatase D (PhoD) superfamily, exhibits unique Ca^2+^‐dependent phospholipase activity. Here, we determined the crystal structure of a PhoD‐type PLD from *S. avermitilis* (SaPLD) at a 2.2‐Å resolution, which shares 86% sequence identity with SchPLD. The structure revealed the conserved Fe‐Ca‐Ca catalytic center characteristic of PhoD enzymes. In addition, we identified novel Ca^2+^ binding sites surrounding the active site pocket. SaPLD exhibited negligible activity in the absence of Ca^2+^ but showed strong activation in the presence of Ca^2+^, consistent with previous observations for SchPLD. The overall structure of SaPLD lacks the C‐terminal α‐helix that covers the active site in *Bacillus subtilis* PhoD, resulting in an expanded hydrophobic cleft suited for bulky phospholipid substrates binding. Molecular dynamics modeling with phosphatidylcholine (PC) indicated that its two oleoyl chains fit well within this cleft, and that the choline head group is accommodated by a distinct cavity formed by Asn217, Leu346, and Asn357. This cavity geometry likely disfavors phosphatidylethanolamine or phosphatidylserine, explaining the preference for PC substrates. These findings provide the first structural insights into the Ca^2+^‐stabilized expanded active site of a PhoD‐type PLD and clarify the molecular basis for its phospholipid specificity.

## 1 Introduction

Phospholipase D (PLD; EC 3.1.4.4) is an enzyme that catalyzes the hydrolysis of phosphodiester bonds of phospholipids (PLs) and produces phosphatidic acid (PA) and a free head group, such as choline. PLDs are distributed in almost all living organisms. Mammalian PLDs are membrane‐bound proteins that are involved in signal transduction. Their product, PA, plays a crucial role in various cellular processes, including cell signaling and vesicle trafficking. Bacteria also possess extracellular (secreted) PLDs, which are presumably involved in host infectivity, for example, cell migration and invasion, as well as phosphate retrieval. Nonetheless, the biological significance of these bacterial PLDs still needs further study.

Bacterial PLDs have been extensively studied for their enzymatic properties, such as substrate specificity and catalytic mechanisms. In previous studies, PLD was shown to catalyze the transesterification of specific PLs in the presence of alcohols [1, 2, 3]. Furthermore, when PLD acts on lysophospholipids (LPLs), it has been reported to produce cyclic PA (cPA), which is presumed to result from a transphosphatidylation involving the release of the polar head groups of the LPLs [1, 4]. Many enzymes exhibiting PLD activity possess one or two copies of the conserved HKD motif (or its variants), which are thought to play a critical role in their catalytic mechanism. These enzymes are classified within the PLD superfamily based on their presumed shared structural features and reaction mechanisms [1, 2, 3].

PLDs derived from soil‐dwelling *Streptomyces* species are widely recognized as valuable biocatalysts in the food, cosmetic, and pharmaceutical industries. Their transphosphatidylation activity enables the efficient synthesis of rare PLs, such as phosphatidylethanolamine, phosphatidylserine or phosphatidylglycerol, as well as unnatural PLs [5] from abundant natural sources like phosphatidylcholine (PC) or lecithin. These PLs can be used as emulsifiers, components of cosmetics, medical formulations, and for liposome preparations [1, 2, 3]. PLDs from *Streptomyces* species, including *S. antibioticus* [6], *S. halstedii* [7], *S. septatus* [8], and the *Streptomyces* sp. PMF strain [9] are among the relatively well‐characterized members of the PLD superfamily. These enzymes share high amino acid sequence identity (~70%) with two conserved HKD motifs (HxKxxxxD), and common biochemical features as metal‐independent activity and high phosphatidyltransferase activity [2, 3, 6, 7, 8, 9]. The crystal structures of PLDs from *S. antibioticus* [10] and *Streptomyces* sp. PMF strain [11] have been reported, elucidating the mechanism of substrate binding and specificity of alcohol transphosphatidylation.

A distinct class of PLDs from *S. chromofuscus* (SchPLD) that exhibits markedly different enzymatic properties has also been reported. SchPLD shares minimal sequence homology with other *Streptomyces* PLDs, lacks canonical HKD motifs but possesses non‐typical HKD‐like sequences (HxKxxxD and HxKxxxxxxxD), and contains metal ions (fully‐occupied Fe^3+^ and partially occupied Mn^2+^/Zn^2+^) [12]. Sequence comparisons indicated that SchPLD is evolutionarily related to enzymes of the bacterial alkaline phosphatase D (PhoD) superfamily. SchPLD exhibits phosphatase activity against various small compounds, such as chromogenic *p*‐nitrophenyl phosphate. Interestingly, the phosphatase/PLD activity of SchPLD is drastically enhanced by the addition of Ca^2+^, while it exhibits almost no transphosphatidylation activity in the presence or absence of Ca^2+^ [12, 13, 14, 15]. Leveraging its Ca^2+^‐dependent activation property, SchPLD has been employed in serum calcium assays. Notably, the PLD method for Ca^2+^ level determination offers the advantage of being less susceptible to interference by magnesium ions and less affected by pH fluctuations compared to the commonly used chelation method employing *o*‐cresolphthalein complexone [16].

In this study, we determined the crystal structure of a PhoD‐type phospholipase D from *S. avermitilis* (SaPLD). SaPLD shares approximately 86% amino acid sequence identity with SchPLD. In the structure of SaPLD, we identified the previously reported Fe^3+^‐Ca^2+^‐Ca^2+^ catalytic center similar to that in *Bacillus subtilis* PhoD (BsPhoD) [17], including three novel Ca^2+^ binding sites. Based on the present structure, the putative mechanism for the PLs binding and selectivity is also discussed.

## 2 Materials and Methods

### 2.1 Expression and Purification of SaPLD

The gene encoding SaPLD, excluding the secretion signal sequence, was synthesized by GenScript Japan Inc. and inserted into the pET‐21a(+) expression plasmid using the *Nde*I and *Hind*III sites, yielding a polypeptide comprising an initiating methionine, residues 44–553 of SaPLD (UniProt ID: Q82AC0), and an additional 13 residues, KLAAALEHHHHHH. The pET21a(+) plasmid harboring the *SaPLD* gene was transformed into *Escherichia coli* BL21(DE3) cells. A single colony was inoculated into 5 mL of LB medium containing 50 μg/mL ampicillin and incubated overnight at 30°C. The preculture was transferred into Overnight Express Instant TB medium (Merck Millipore, MA, USA) supplemented with 1% (v/v) glycerol and 50 μg/mL ampicillin and cultivated at 28°C for 23 h. The cells were harvested via centrifugation and resuspended in buffer A (10 mM Tris‐HCl [pH 7.5] and 0.3 M NaCl). The cells were disrupted via sonication, and the lysate was clarified via centrifugation. The supernatant was added to a Ni‐Sepharose 6 Fast Flow column (75 mL bed volume; Cytiva, Uppsala, Sweden) equilibrated with buffer A. The column was washed sequentially with five column volumes (CV) of buffer A and five CV of 12.5% buffer B (10 mM Tris‐HCl [pH 7.5], 0.3 M NaCl, and 0.4 M imidazole). The bound SaPLD was then eluted with a linear gradient from 12.5% to 100% buffer B over 10 CV. The eluate was dialyzed against 25 mM Tris‐HCl (pH 7.0) and subsequently applied to a CM Fast Flow column (50 mL; Cytiva) equilibrated with buffer C (25 mM Tris‐HCl [pH 7.0]). After washing with 4 CV of buffer C, the bound SaPLD was eluted with a linear gradient from 0% to 100% buffer D (25 mM Tris‐HCl [pH 7.0], 0.5 M KCl) over 20 CV. Fractions containing SaPLD were pooled and concentrated using an Amicon Ultra‐15 centrifugal filter device (30 kDa MWCO; Merck Millipore). The resulting sample was desalted on a PD‐10 column (Sephadex G‐25; Cytiva) equilibrated with 10 mM Tris–HCl (pH 8.0), followed by a final concentration using the same device. The purified enzyme was stored at 4°C until use.

### 2.2 Enzyme Assay

All enzyme assays were conducted using a Hitachi 7180 automatic analyzer (Hitachi, Tokyo, Japan). Unless otherwise specified, all chemicals and reagents were purchased from Fujifilm Wako Pure Chemical Corporation (Tokyo, Japan). Reaction mixture I (40 mM Tris–HCl [pH 8.0]; 0, 10, or 100 mM Ca acetate; and 5 mM L‐α‐PC) was used to hydrolyze the phosphodiester bonds in PC. Reaction mixture II (40 mM Tris–HCl [pH 8.0], 24 mM EDTA [pH 8.0], 0.6 mM 4‐aminoantipyrine [4‐AA], 0.008% [w/v] *N*,*N*‐bis[4‐sulfobutyl]‐3‐methylaniline [TODB], 3.6 U/mL peroxidase, and 1.2 U/mL choline oxidase [COD; Nagase Diagnostics Co. Ltd., Shizuoka, Japan]) was used for the oxidation of choline followed by the oxidative coupling of 4‐AA and TODB. A 25 mM PC stock solution was prepared in 5% (v/v) Triton X‐100. The purified SaPLD was diluted in 10 mM Tris‐HCl (pH 8.0) containing 0.05% (w/v) bovine serum albumin and 0.1% (w/v) Triton X‐100. Each assay contained 40 μL of reaction mixture I and 4 μL of the appropriately diluted enzyme solution, which was incubated for 5 min at 37°C. Subsequently, 200 μL of reaction mixture II was added and incubated for an additional 5 min at 37°C, after which the absorbance at 546 nm was measured. The reagent blank was prepared using the dilution buffer only. One unit (U) of SaPLD activity was defined as the amount of enzyme catalyzing the hydrolysis of 1 μmol of PC per min under the above assay conditions (*ε*_546_ = 36 mM^−1^ cm^−1^). Protein concentrations were determined using the Bradford protein assay. Commercially available SchPLD (PLD II; Nagase Diagnostics Co. Ltd., Shizuoka, Japan) was assayed under identical conditions for comparison.

### 2.3 Crystallization

Initial crystallization screening was performed using Crystal Screen 1 and 2 (Rigaku, Tokyo, Japan), Wizard Classic 1 and 2 (Emerald BioStructures, WA, USA), and ProPlex MD1‐38 (Molecular Dimensions, OH, USA). Diffraction‐quality crystals were obtained using the sitting‐drop vapor‐diffusion method at 20°C using a reservoir solution containing 0.1 M Na cacodylate buffer (pH 5.5), 0.1 M Ca acetate, and 13% (w/v) PEG 8000. The crystallization droplet was formed by mixing 1 μL of the enzyme solution (12 mg/mL) and 1 μL of the reservoir solution and equilibrated against 100 μL of the reservoir solution in 96‐well Compact Clover Crystallization Plates (Rigaku, Tokyo, Japan). Single crystals with purple color appeared in the droplet in 3 days and grew to a maximum dimension of 0.1 × 0.2 × 0.4 mm. The crystals were briefly soaked in the reservoir solution containing 30% ethylene glycol and flash‐cooled in liquid nitrogen.

### 2.4 x‐Ray Diffraction Study and Model Refinement

x‐ray diffraction experiments were performed using synchrotron radiation on beamline AR NW‐12A at the Photon Factory (Tsukuba, Japan). The raw diffraction data were indexed, scaled, and merged using the HKL2000 package [18]. The structure was determined using the molecular replacement method with the program MOLREP [19]. The atomic model of BsPhoD (Protein Data Bank [PDB] accession code 2YEQ) [17] served as the search model. Model refinement was performed using REFMAC5 [20] and Phenix [21]. Non‐crystallographic symmetry restraints were not applied. Manual model fitting was conducted using the program Coot [22]. The stereochemical qualities of the final refined models were assessed using MolProbity [23]. The data collection and refinement statistics are summarized in Table 1.

**TABLE 1:** Crystallographic parameters and model refinement statistics.

*Data collection*	
Beamline	Photon Factory AR‐NW12A
Wavelength (Å)	1.0000
Temperature (K)	100
Detector	DECTRIS PILATUS 2M
Space group	*P*2_1_2_1_2_1_
Unit‐cell parameters (Å)	*a* = 90.9, *b* = 156.8, *c* = 163.8
Resolution (Å)^a^	50–2.20 (2.24–2.20)
Unique reflections	119 057
*R*_meas_ ^a^, ^b^	0.109 (0.642)
Mean *I*/*σ* (*I*)^a^	16.7 (2.1)
Completeness (%)^a^	99.7 (97.5)
Multiplicity^a^	6.5 (6.3)
Wilson *B* value (Å^2^)	32.8
*Model refinement*	
*R*_work_/*R*_free_ ^c^, ^d^	0.177/0.211
Number of atoms	
All/polypeptide chains/ligands/waters	16 553/15739/44/770
Average *B*‐factors (Å^2^)	
All/polypeptide chains/ligands/waters	43.4/43.6/33.6/39.9
RMSD from ideal	
Bond lengths (Å)/bond angles (°)	0.004/0.66
Ramachandran plot	
Favored/allowed/outliers (%)	95.7/4.3/0.0

^a^ The values shown in parentheses are for the outermost resolution shell.

^b^ *R*_meas_ = Σ_h_Σ_*i*_|*I*_h,*i*_ − <*I*_h_>|/Σ_h_Σ_*i*_ *I*_h,*i*_, where <*I*_h_> is the mean intensity for a set of equivalent reflections.

^c^ *R*_work_ = Σ|*F*_obs_—*F*_calc_|/Σ *F*_obs_ for 95% of the reflection data used in the refinement. *F*_obs_ and *F*_calc_ are the observed and calculated structure factor amplitudes, respectively.

^d^ *R*_free_ is the equivalent of *R*_work_, except that it was calculated for a randomly chosen 5% of reflections, which were excluded from refinement.

### 2.5 Other Software

Molecular drawings were prepared using PyMol (Schrödinger LLC, USA). Multiple sequence alignment was performed using ClustalX [24], and visualized with secondary structure elements using ESPript ver. 3 [25]. The possible conformation of the bound PC (1,2‐dioleoyl‐*sn*‐glycero‐3‐phosphocholine [PDBeChem code, PCW]) at the SaPLD active site cleft was inferred using the program phenix.dynamics (simple model perturbation) implemented in the Phenix package [21].

## 3 Results and Discussion

### 3.1 Ca^2+^‐Dependent Enzyme Activity of SaPLD

The enzymatic activity of SaPLD was measured using a coupled assay with COD and peroxidase using a previously reported method [26]. The reaction product, free choline, was quantified based on the formation of a quinoneimine dye, which was monitored spectrophotometrically by measuring the absorbance at 546 nm. To evaluate the Ca^2+^ dependency of SaPLD activity, assays were performed in the absence and presence of Ca acetate (10 mM). In addition, since the crystal structure of SaPLD was determined in the presence of 100 mM Ca acetate, enzyme activity was also examined under this condition (100 mM Ca acetate). As summarized in Table 2, SaPLD exhibited negligible activity in the absence of Ca acetate, whereas substantial activity was detected with 10 mM Ca acetate and further increased by approximately 1.4‐fold with 100 mM Ca acetate. A similar enhancement at 100 mM Ca acetate was also observed for SchPLD. The overall activity level of SaPLD was comparable to that of the well‐characterized SchPLD. These results are consistent with the Ca^2+^‐dependent enzymatic properties previously reported for SchPLD [13, 14, 15] and demonstrate, for the first time, that its activity can be further stimulated by high Ca^2+^ concentrations (100 mM). In nature, these enzymes are likely activated by the Ca^2+^ ions that are ubiquitously present in soil.

**TABLE 2:** Enzyme activities of SaPLD and SchPLD with varying Ca acetate concentrations (*n* = 5).

	Specific activity (U/mg)		
	0 mM Ca acetate	10 mM Ca acetate	100 mM Ca acetate
SaPLD	1.37 ± 0.27 (1.58)	86.7 ± 0.20 (100.0)	120.2 ± 0.46 (138.6)
SchPLD	3.36 ± 0.35 (3.70)	91.0 ± 0.57 (100.0)	106.6 ± 0.81 (117.1)

*Note:* The values in parentheses refer to the enzyme activities (%) relative to that in the presence of 10 mM Ca acetate.

### 3.2 Overall Structure of SaPLD and Structural Comparison

**FIGURE 1:**
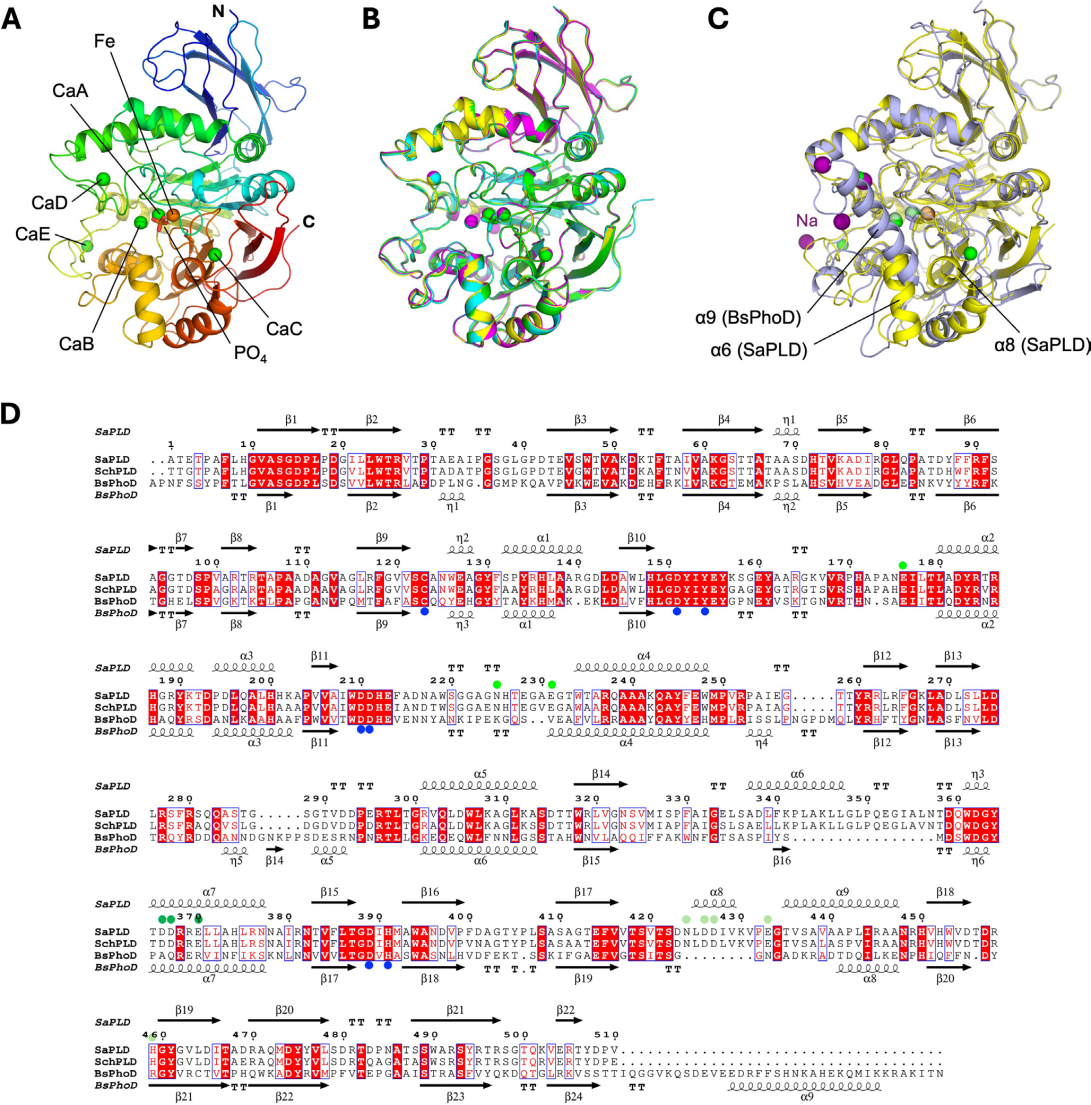
Structure of SaPLD and comparison to that of BsPhoD. (A) Ribbon representation of the overall structure of SaPLD. The model is colored according to the sequence using a rainbow color ramp from the N‐terminus (blue) to the C‐terminus (red). The bound Ca^2+^ (CaA‐CaE), Fe^3+^, and phosphate ions are indicated. (B) Superimposition of the four SaPLD monomers in the asymmetric unit, which are colored yellow, cyan, green, and magenta. (C) Superimposition of SaPLD (yellow) and BsPhoD (light blue). The bound metals in SaPLD are shown in the same color scheme in (A). The bound Fe^3+^, Ca^2+^, and Na^+^ ions in BsPhoD are colored light orange, light green, and purple, respectively. The differences in secondary structure elements between SaPLD and BsPhoD are indicated. (D) Amino acid sequence alignment between SaPLD, SchPLD, and BsPhoD. Amino acid numbering is presented for SaPLD. White letters on a red background indicate fully conserved residues. Red letters indicate partially conserved residues. The conserved residues for Fe‐CaA‐CaB binding at the catalytic site are indicated by the blue circle. The residues for CaC, CaD, and CaE binding conserved in SaPLD and SchPLD are indicated by light green, green, and dark green circles, respectively. Secondary structure elements are depicted for SaPLD (top) and for BsPhoD (bottom).

We have determined the crystal structure of SaPLD at a 2.2‐Å resolution. The final refined model in the asymmetric unit consists of four independent (symmetry‐unrelated) SaPLD monomers and 4 Fe^3+^, 4 PO_4_^3−^, 20 Ca^2+^, and 770 water molecules, with *R*/*R*_free_ factors of 0.177/0.211. Each monomer exhibits a typical β‐sandwich core created by opposing antiparallel β‐sheets and flanking α‐helices (Figure 1A). The four SaPLD monomers in the asymmetric unit are very well‐superimposed with each other with a main‐chain root mean square deviation (RMSD) of ~0.5 Å. The distinct conformations are not observed, including loop structures on the molecular surface; thus, the inter‐monomer crystal contacts do not affect both the overall and local conformations of SaPLD (Figure 1B). The DALI search [27] demonstrated that the structure of SaPLD is the most homologous to that of BsPhoD (PDB code, 2YEQ [17]) with an RMSD of 2.0 Å and a *Z* score of 48.6 for the main‐chain Cα atoms (36% sequence identity for the fit regions) (Figure 1C). It is noted that no PLD activity has been reported for BsPhoD. There is a notable structural difference between SaPLD and BsPhoD: the long C‐terminal α‐helix (α9 of BsPhoD) is completely missing in the structure of SaPLD, and the gap due to the lack of this α9 is partially filled by the α6 and α8 of SaPLD, which correspond to the SaPLD/SchPLD‐specific insertion sequence, and are not present in BsPhoD (Figure 1C,D). A previous study elucidated that the α9 of BsPhoD fully covers the active site cleft and is essential for the metal retention and catalytic activity of BsPhoD [17]. Due to the lack of α9, SaPLD exhibits an expanded hydrophobic active site cleft, which might be suitable for the binding of bulky and hydrophobic PLs to the active site, as discussed later. Interestingly, in the AlphaFold model of SaPLD (AlphaFold database ID, AF‐A0A4D4M3D9‐F1‐v6) [28], α6 protrudes outward from the active site, exposing a cluster of five Leu residues to the solvent (Figure S1). This suggests that α6 may possess intrinsic mobility and could help capture substrates and guide them into the cleft. Similar structural mobility within the active site cleft has been reported in the crystallographic study of cholesterol esterase [29].

### 3.3 Identification of Multiple Ca^2+^ Binding Sites Around the Active Site

**FIGURE 2:**
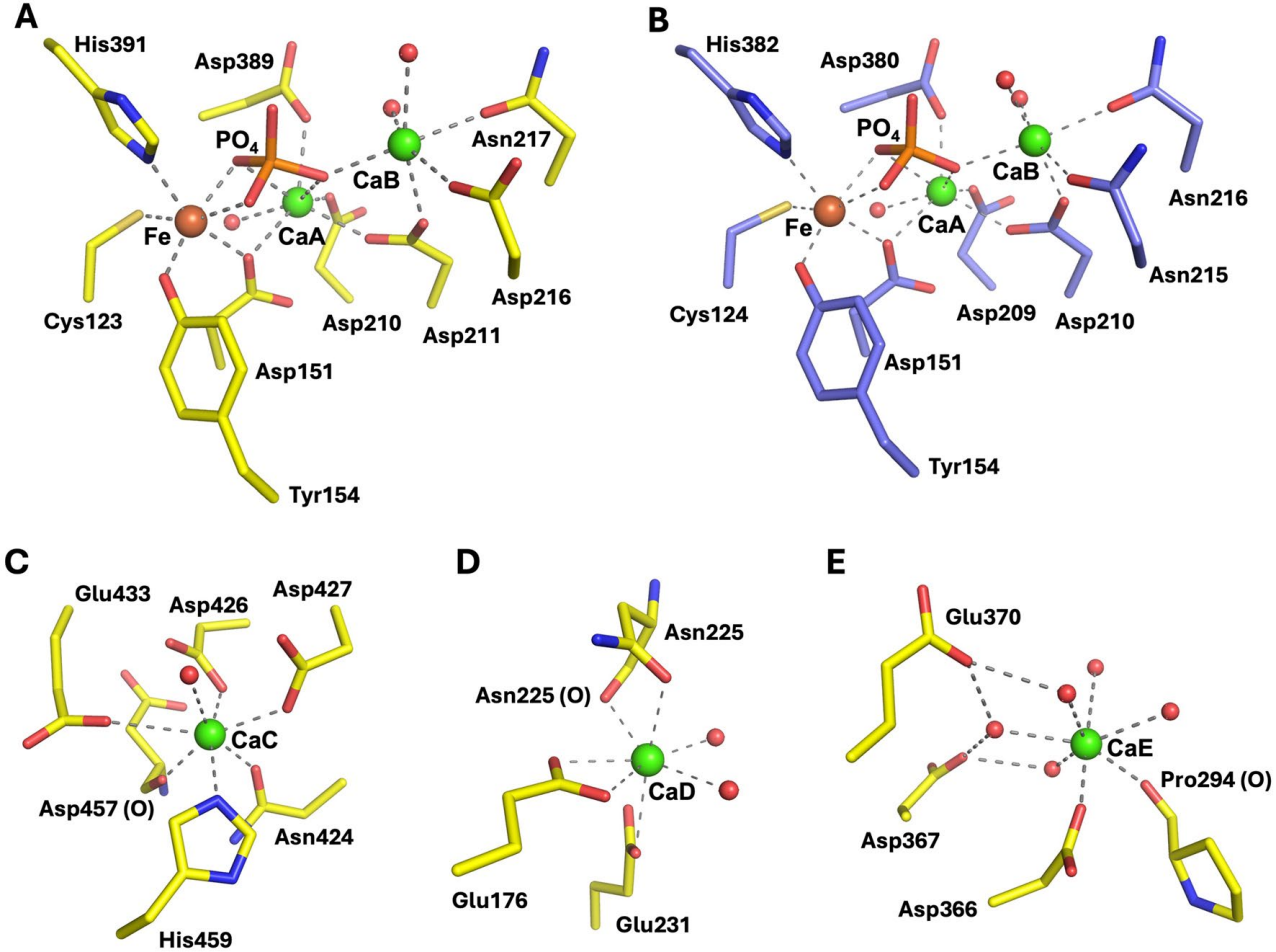
Ca^2+^ binding sites of SaPLD. (A) Fe‐CaA‐CaB‐PO_4_ cluster in the active site of SaPLD and nearby residues for metal coordination (dashed lines). (B) The active site structure of BsPhoD is very similar to that of SaPLD. Novel Ca^2+^ binding site structures: CaC (C), CaD (D), and CaE (E). The solvent molecules coordinated to Ca^2+^ are also shown in red small spheres.

During model refinement, strong residual electron density was observed within the active site pocket, which was surrounded by nine amino acid side chains. The electron density closely matched the Fe^3+^‐Ca^2+^‐Ca^2+^‐PO₄ (Fe‐CaA‐CaB‐PO₄) cluster reported in BsPhoD [17]. The atomic *B*‐factors of these metal ions were comparable to those of neighboring residues and waters with full occupancy (Figure 2A,B, and Table S1). The conserved Fe‐CaA‐CaB‐PO₄ cluster indicates a shared catalytic mechanism between SaPLD and BsPhoD. In addition to this Fe‐CaA‐CaB‐PO₄ cluster, three extra Ca^2+^ ions (CaC, CaD, and CaE) were identified around the active site (Figure 2C–E). CaC lies near the active site cleft approximately 12 Å from Fe^3+^ and exhibits typical 7‐fold coordination involving Asn424, Asp426, Asp427, Glu433, His459, the main‐chain oxygen of Asp457, and one water molecule. These residues are conserved in SchPLD but absent in BsPhoD due to the lack of α‐helix α8 (Figure 1D). CaD is located on the opposite side of the cleft, approximately 16 Å from Fe^3+^, and is also sevenfold coordinated by Glu176, Glu231, Asn225, the main‐chain oxygen of Asn225, and two water molecules. In BsPhoD, only Glu176 is conserved, whereas Glu231 and Asn225 are not (Figures 1D and 2D); a Na^+^ ion occupies the equivalent site. Both CaC and CaD lie in proximity to the active site and likely contribute to the stabilization of the active site cleft. In contrast, CaE is located on the molecular surface between helices α5 and α7, approximately 27 Å from the active site. It shows sixfold or sevenfold coordination with Asp366, the main‐chain oxygen of Pro294, and water molecules hydrogen‐bonded to Glu370 and Asp367. These acidic residues are conserved in SchPLD but not in BsPhoD. Compared with CaC and CaD, CaE is located farther from the active site and exhibits higher *B*‐factors, suggesting that it may represent a crystallization artifact. All Ca^2+^ ions bound to SaPLD are solvent‐accessible and likely undergo reversible association and dissociation, consistent with the reduced enzyme activity observed under Ca^2+^‐free conditions (Table 2). The observed enhancement of SaPLD and SchPLD activities in the presence of 100 mM Ca acetate suggests that the bound Ca^2+^ ions do not interfere with substrate binding or catalysis.

### 3.4 Insights Into PL Binding and Head Group Selectivity

**FIGURE 3:**
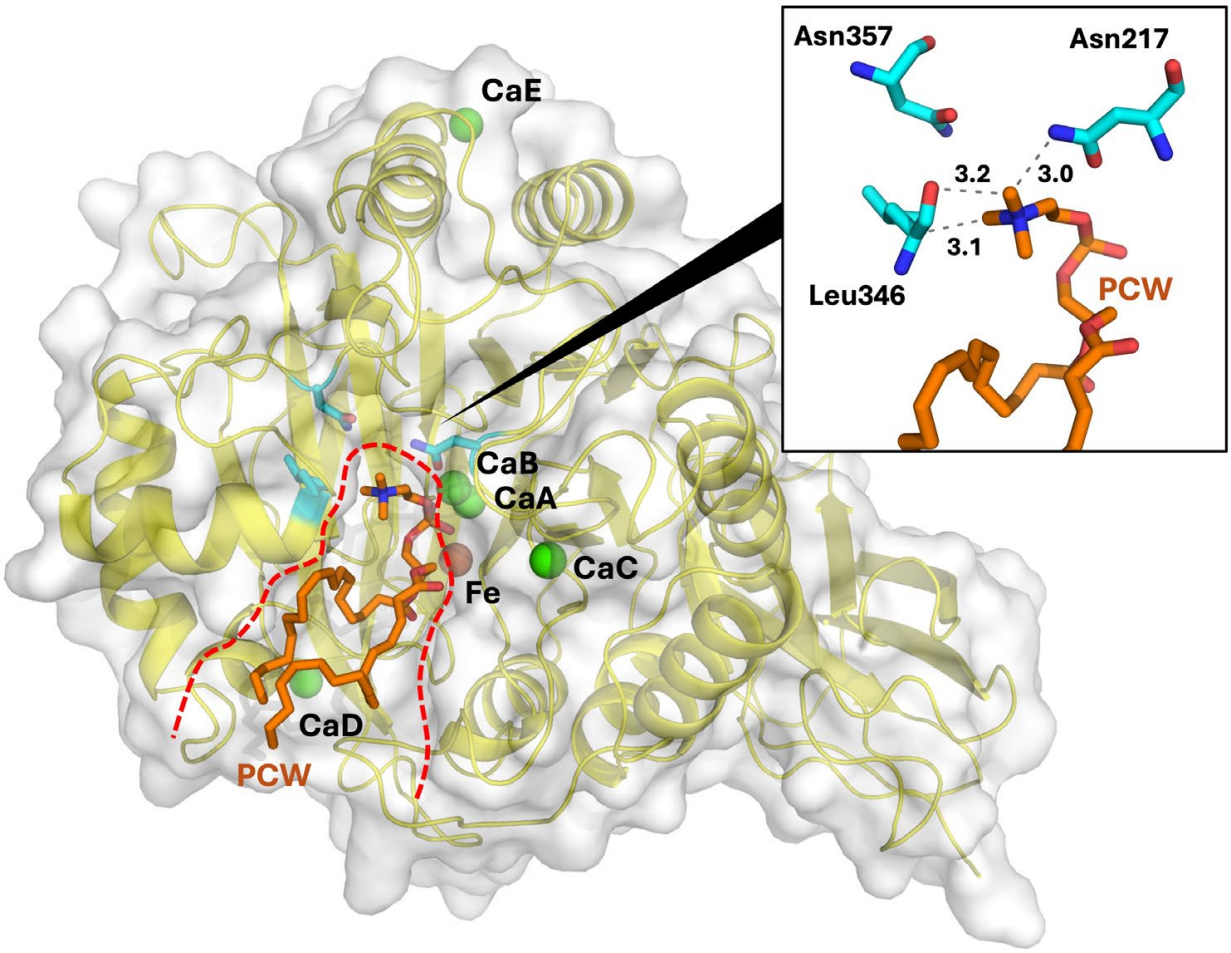
Presumed binding model of phosphatidylcholine (PCW) to the expanded active site cleft of SaPLD. The monomer structure of SaPLD is shown by overlaying the transparent molecular surface and ribbon diagram in yellow. The expanded active site cleft is roughly indicated by the red dotted lines. The close‐up view of the choline moiety of PCW located at the cavity surrounded by Asn217, Leu346, and Asn357 in cyan is also shown. The distances between the methyl groups and nearby atoms are also shown (Å).

To generate a putative conformation of the bound substrate (PCW) at the active site, we employed simple molecular dynamics simulations using phenix.dynamics implemented in the Phenix program. This approach was used to relieve steric clashes while preserving the Ca^2+^–phosphate coordination, yielding a structurally reasonable substrate‐binding model. The phosphate moiety of PCW was superimposed onto the bound phosphate observed in the present structure, and simulations were performed with default settings, resulting in a plausible positioning of the oleic acid chains within the expanded active‐site cleft without steric clashes (Figure 3). It was found that the expanded active site cleft of SaPLD had a size suitable for accommodating the two oleic acid chains of the substrate. The model also showed that the choline moiety of PCW is located at the cavity created by Asn217, Leu346, and Asn357, and the methyl groups of the choline are well‐fitted to the cavity with distances of 3.0–3.2 Å from the side‐chain atoms of Asn217 and main‐chain atoms of Leu346 (Figure 3). Asn357 lies deeper in this cavity, ~4.5 Å from the choline methyl group. This cavity is absent in BsPhoD, where about 20 amino acid residues including Leu346 and Asn357 are deleted, but are conserved in SchPLD (Figure 1D). The cavity geometry of SaPLD may disfavor a substrate lacking the three methyl groups of choline (phosphatidylethanolamine), or that bearing a branched carboxyl‐containing head group (phosphatidylserine). This model is consistent with previous reports showing that SchPLD displays high activity toward PC but lower activity toward phosphatidylethanolamine and phosphatidylserine [30]. To clarify the mechanism of head group selectivity, mutational studies targeting Asn217/346 are necessary and remain a future challenge. Collectively, the finding that PCW fits well into the expanded active site pocket of SaPLD provides a structural rationale for the adaptation to PC binding and PLD activity in the PhoD‐related enzyme.

## Author Contributions

**Yoshiaki Yasutake:** conceptualization, formal analysis, investigation, writing – original draft. **Tatsuya Hirata:** data curation, formal analysis, investigation, writing – review and editing. **Shunsuke Nomura:** investigation, writing – review and editing. **Kenji Konishi:** investigation, writing – review and editing. **Kazunari Yoneda:** investigation, writing – review and editing. **Shin‐ichi Sakasegawa:** conceptualization, data curation, formal analysis, investigation, writing – original draft. **Haruhiko Sakuraba:** conceptualization, supervision, data curation, formal analysis, writing – original draft.

## Funding

The authors have nothing to report.

## Conflicts of Interest

The authors declare no conflicts of interest.

## Supporting information


**Table S1:** The bound metal ions, coordinating ligands, and their mean *B* factors in the SaPLD structure (chains A–D).
**Figure S1:** Structural comparison of SaPLD between the crystallographic model (this study) and the AlphaFold model (AF‐A0A4D4M3D9‐F1‐v6).

## Data Availability

Atomic coordinates and structural factors were deposited in the PDB under accession code 9UYA. Other data generated and/or analyzed during this study are available from the corresponding authors upon reasonable request.
