# Supplementary material for: x‐Ray Structure of Streptomyces avermitilis Phospholipase D Reveals a Ca2+‐Stabilized Expanded Active‐Site Cleft Adapted for Phospholipid Binding
Source: Proteins. 2026 Apr 30;94(9):1543–50. doi: 10.1002/prot.70142 (PMC13435022; doi:10.1002/prot.70142)
Supplement: Supplementary file 1 [file PROT-94-1543-s001.pdf]

Supplementary information:

X-ray structure of *Streptomyces avermitilis* phospholipase D reveals a Ca^{2+}-stabilized expanded active-site cleft adapted for phospholipid binding

Yoshiaki Yasutake[a*], Tatsuya Hirata[b], Shunsuke Nomura[c], Kenji Konishi[b], Kazunari Yoneda[d], Shin-ichi Sakasegawa[e], Haruhiko Sakuraba[f]

[a] Biomanufacturing Process Research Center, National Institute of Advanced Industrial Science and Technology (AIST), Sapporo 062-8517, Japan.

[b] Nagase Diagnostics Co., Ltd., Shizuoka 410-2321, Japan.

[c] Asahi Kasei Pharma Corporation, Shizuoka 410-2321, Japan.

[d] Department of Food and Life Sciences, School of Agriculture, Tokai University, Kumamoto 861-2205, Japan.

[e] Biomanufacturing Process Research Center, AIST, Tsukuba 305-8566, Japan.

[f] Department of Applied Biological Science, Faculty of Agriculture, Kagawa University, Kagawa 761-0795, Japan.

*Corresponding author: Yoshiaki Yasutake

Tel.: +81 50-3522-8972; Email: y-yasutake@aist.go.jp

Supplementary Table S1. The bound metal ions, coordinating ligands, and their mean *B*-factors in the SaPLD structure (Chains A to D)

Metal ions and coordinating atoms	Mean *B*-factor values
Fe	30.4 ± 3.1
Cys123 SG	28.7 ± 4.1
Asp151 OD2	29.5 ± 2.5
Try154 OH	30.7 ± 5.6
His391 NE2	30.1 ± 3.8
PO4 O3	31.7 ± 5.1
PO4 O4	30.2 ± 4.6
CaA	29.8 ± 3.1
Asp151 OD2	29.5 ± 2.5
Asp210 OD2	27.6 ± 4.1
Asp211 OD1	29.4 ± 3.2
Asp389 OD1	28.2 ± 1.7
PO4 O4	30.2 ± 4.6
PO4 O2	33.2 ± 1.0
Water (A1232/B1245/C1213/D1207)	27.3 ± 1.7
CaB	30.5 ± 3.1
Asp211 OD2	31.4 ± 3.5
Asp216 OD1	30.6 ± 3.3
Asn217 OD1	30.4 ± 2.0
PO4 O2	33.2 ± 1.0
Water (A1151/B1143/C1123/D1136)	29.3 ± 2.0
Water (A1228/B1278/C1322/D1231)	32.5 ± 3.9
CaC	33.3 ± 7.1
Asn424 OD1	33.7 ± 7.1
Asp426 OD1	37.3 ± 6.7
Asp427 OD2	37.6 ± 6.4
Glu433 OE1	41.4 ± 5.1
Asp457 O	35.1 ± 7.9
His459 NE2	36.5 ± 8.8
Water (A1173/B1122/C1102/-)	39.7 ± 8.1
CaD	40.6 ± 7.4
Glu176 OE1	40.9 ± 8.8

Glu176 OE2	40.6 ± 8.9
Glu231 OE2	45.3 ± 7.6
Asn225 O	38.8 ± 7.1
Asn225 OD1	37.0 ± 6.5
Water (A1211/B1139/C1256/D1182)	36.6 ± 4.9
Water (A1247/B1288/C1284/-)	37.6 ± 5.1
CaE	43.3 ± 7.2
Pro294 O	44.3 ± 7.3
Asp366 OD2	40.5 ± 7.5
Water (A1103/B1120/C1121/D1101)	40.7 ± 6.1
Water (A1121/B1149/C1137/D1110)	33.7 ± 6.1
Water (A1150/B1256/C1187/D1180)	37.3 ± 5.3
Water (A1259/B1300/C1311/D1230)	37.1 ± 3.0
Water (-/-/C1288/-)	42.5
Asp367 OD1*	34.3 ± 6.3
Glu370 OE2*	39.4 ± 6.4

Asterisks represent the residues interacting with CaE via water molecules (see Fig. 2).

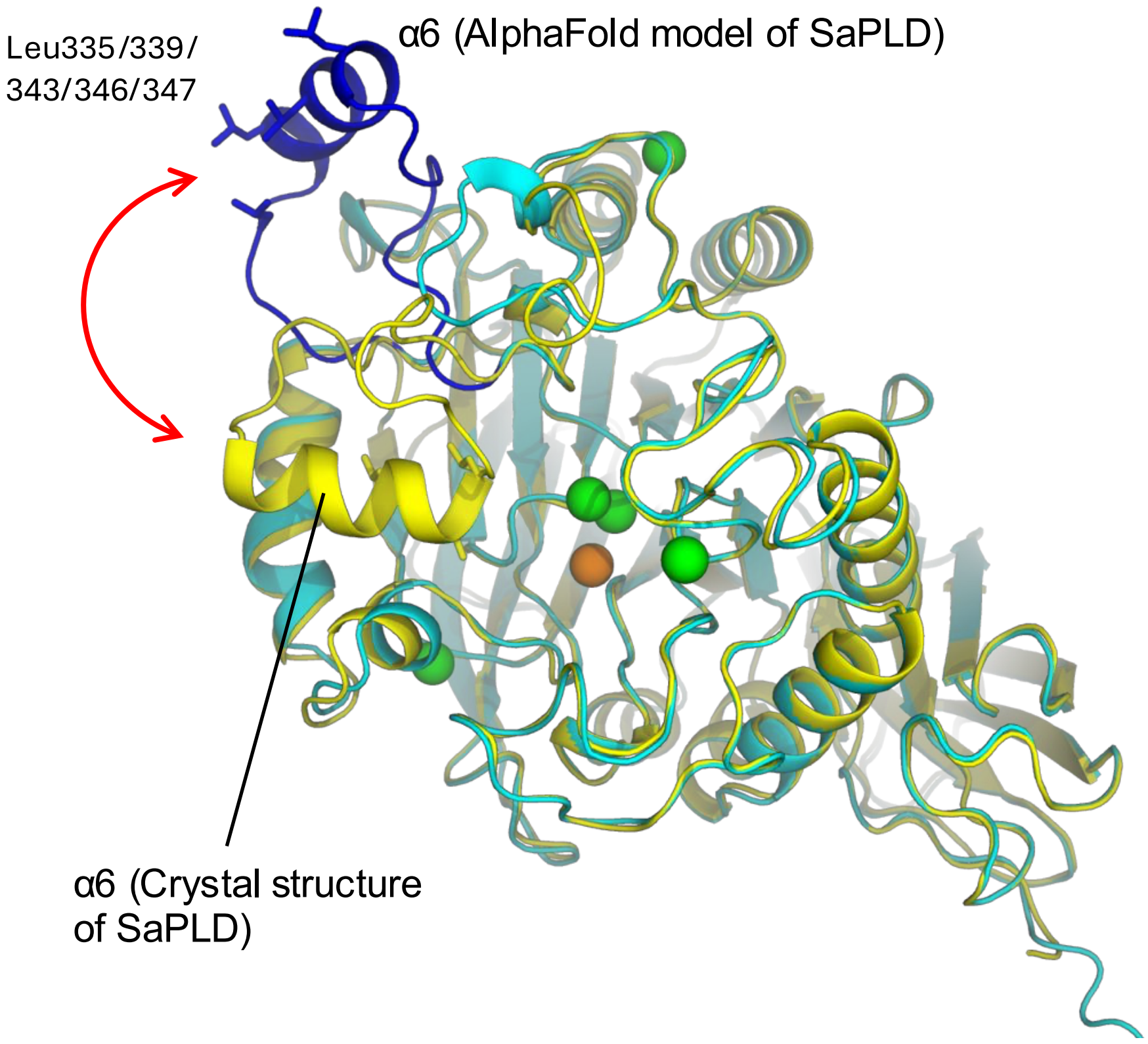

Supplementary Fig. S1. Structural comparison of SaPLD between the crystallographic model (this study) and the AlphaFold model (AF-A0A4D4M3D9-F1-v6). The view is approximately the same as in Fig. 3. The crystal structure of SaPLD determined in this study is shown in yellow. The AlphaFold model is shown in cyan, with the region that differs markedly from the crystal structure, including α6, highlighted in blue. Leu residues forming the cluster are shown as sticks and labeled. The potential mobility of α6 is indicated by a red arrow.
